# Design, Implementation and Characterization of a Quantum-Dot-Based Volumetric Display

**DOI:** 10.1038/srep08472

**Published:** 2015-02-16

**Authors:** Ryuji Hirayama, Makoto Naruse, Hirotaka Nakayama, Naoya Tate, Atsushi Shiraki, Takashi Kakue, Tomoyoshi Shimobaba, Motoichi Ohtsu, Tomoyoshi Ito

**Affiliations:** 1 Graduate School of Engineering, Chiba University, 1-33 Yayoi-cho, Inage-ku, Chiba 263-8522, Japan; 2 Photonic Network Research Institute, National Institute of Information and Communications Technology, 4-2-1 Nukui-kita, Koganei, Tokyo 184-8795, Japan; 3 Graduate School of Information Science and Electrical Engineering, Kyushu University, 744 Motooka, Nishi-ku, Fukuoka 819-0395, Japan; 4 Department of Information and Computer Engineering, Kisarazu National College of Technology, 2-11-1 Kiyomidai-higashi, Kisarazu, Chiba 292-0041, Japan; 5 Graduate School of Engineering, The University of Tokyo, 2-11-16 Yayoi, Bunkyo-ku, Tokyo 113-8656, Japan

## Abstract

In this study, we propose and experimentally demonstrate a volumetric display system based on quantum dots (QDs) embedded in a polymer substrate. Unlike conventional volumetric displays, our system does not require electrical wiring; thus, the heretofore unavoidable issue of occlusion is resolved because irradiation by external light supplies the energy to the light-emitting voxels formed by the QDs. By exploiting the intrinsic attributes of the QDs, the system offers ultrahigh definition and a wide range of colours for volumetric displays. In this paper, we discuss the design, implementation and characterization of the proposed volumetric display's first prototype. We developed an 8 × 8 × 8 display comprising two types of QDs. This display provides multicolour three-type two-dimensional patterns when viewed from different angles. The QD-based volumetric display provides a new way to represent images and could be applied in leisure and advertising industries, among others.

Volumetric displays are one of the most interesting technologies for three-dimensional (3D) display and human–computer interactions and thus have received significant research attention^1^,^2^. Unlike conventional two-dimensional displays, a volumetric display has a physical 3D architecture that enables 3D images to be observed from any surrounding viewpoint. Various volumetric displays have been proposed previously in the literature; e.g. arrays of light-emitting diodes arranged in a 3D layout or arrays of strings on which an image is projected, where the strings serve to scatter light^3^. However, to date, the presence of occlusion, which is the distortion of a part of the image viewed from a certain direction because of the scattering from the electrical wiring or other scattering centres, has led to a significant degradation in the quality of the 3D images (Fig. 1a).

In this paper, we present a volumetric display that eliminates occlusion by using quantum dots (QDs) arranged in a 3D manner. In our device, the light-emitting elements are formed by the QDs. Irradiation by an external invisible or visible light source excites the QDs and generates spontaneous emission; this in turn creates patterns that can be simultaneously observed from various viewing angles. Figure 1b schematically shows the concept of the volumetric display based on QD luminescence, where three different patterns (‘X', ‘Y', and ‘Z') are observed from different viewing directions without occlusion. In addition, for simplicity, we employ other patterns in the experimental demonstrations while maintaining essential operating principles.

In addition to eliminating the electrical wiring, our QD-based volumetric display has various other unique features. The emission spectrum of the QDs depends on their size and constituent material; thus, various colours can be achieved^4^. Small-diameter QDs emit light with short wavelengths, whereas large-diameter QDs emit light with long wavelengths. Compared with other luminescence materials, the QDs exhibit higher quantum efficiencies and narrower emission spectra^5^. The 2D displays using these features of the QDs have attracted considerable attention^6^,^7^,^8^ and have previously been used in practical applications. Notably, these favourable properties are also important for the 3D volumetric display applications. Moreover, when the different-sized QDs are closely packed, optical energy transfer occurs among them because of the nanometre-scale near-field interactions^9^. Although this energy-transfer mechanism has not been used for the experiment reported herein, it will be exploited to provide additional functionality in future. In the sections below, we describe the design, fabrication and characterization of the proposed QD-based volumetric display.

## Results

### Design

In this study, we developed a QD-based volumetric display that exhibits three different images that may be viewed simultaneously from three orthogonal viewpoints. Let a voxel be denoted by *V_ijk_*, where *i*, *j* and *k* take the values 1,…,*N*, giving a total of *N*^3^ voxels. The value of *V_ijk_* is specified by the type of the QDs present in the corresponding voxel and is zero if there are no QDs. Let the type of a QD be given by {*S*_1_,*S*_2_,…,*S_M_*}, where *M* is the number of the QD types. The images observed from the *x*, *y* and *z* axes are given by 
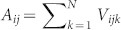
, 
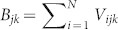
 and 
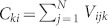
, respectively. Note that here, the summation does not necessarily imply arithmetic addition in the usual sense; rather, it implies the addition of the light spectra and is recognized as a given colour by the human eye. For instance, if *S*_1_ is red and *S*_2_ is green, the sum *S*_1_ + *S*_2_ is yellow. Furthermore, as shown below, in our experimental prototype, the sequence of *S_i_* as observed from a viewpoint could be important depending on the absorption and transmission coefficients of each voxel.

For our experimental prototype, we selected *N* = 8, and *V_ijk_* could take values of 0, red or green. Therefore, because red + green = yellow, we get a total of four colours (red, green, yellow and no colour or transparent).

**Figure 2:**
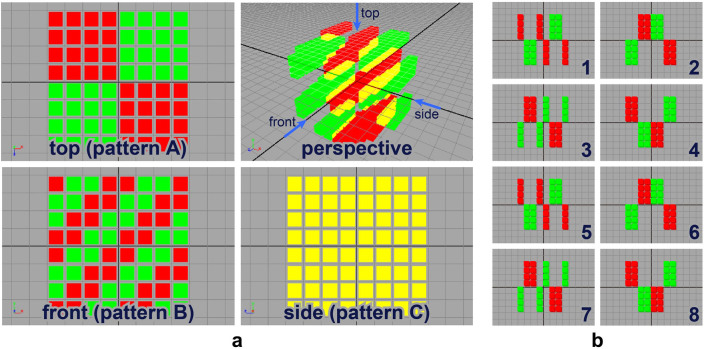
Design of a QD-based volumetric-display prototype. (a) QD-based volumetric display viewed from different angles. The prototype comprises 8 × 8 × 8 voxels. (b) Design data for the QD-based volumetric display consisting of eight layers.

To completely convey the capacity of our QD-based volumetric display, we designed the voxels for (i) the three resulting images (*A*, *B* and *C*) to differ from each other and (ii) yellow to appear only in one of the three images. Figure 2a schematically shows the design in which patterns *A* (top-left panel), *B* (lower-left panel) and *C* (lower-middle panel) differ from each other; only pattern *C* contains yellow. The design of each layer is presented in Fig. 2b. Although the formula described above does not enable three arbitrary patterns to be created without any artefact, Nakayama *et al.* have recently demonstrated a novel algorithm for the design of voxels that provide arbitrary numbers and arbitrary images by distributing the effects of the artefacts^10^. This method may be applied to the proposed QD-based volumetric display.

### Implementation

**Figure 3:**
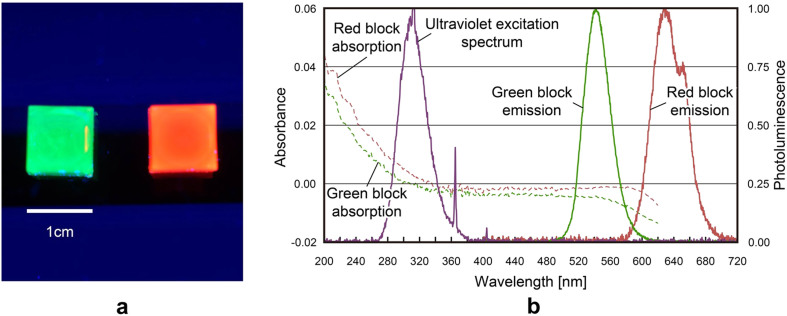
Solid blocks containing QDs. (a) Photographs of the red and green blocks when excited by ultraviolet irradiation. (b) Normalized absorption and emission spectra of the red and green blocks. The spectrum of the ultraviolet excitation source used to excite the QDs is also shown.

Figure 3a shows a photograph of the solid blocks containing the QDs used as the light-emitting voxels. In this photograph, the blocks are exposed to ultraviolet radiation with 302 nm wavelength (AS ONE, ‘MID-170', Transilluminator). The blocks containing QDs are made by encapsulating a certain amount of the QDs in a clear polydimethylsiloxane (PDMS) polymer. (Details are given in the Methods section.) The absorption spectra of the QDs are barely affected by the inclusion in the polymer because PDMS is a heat-curable resin that is transparent at visible light wavelengths. Because of its transparency, PDMS is used in various applications^11^,^12^. We made two types of blocks. When irradiated by ultraviolet light, the QDs in one block emit red light, whereas those in the other block emit green light; we refer to these blocks as the ‘red block' and the ‘green block', respectively. They have dimensions of approximately 1 cm × 1 cm × 1 cm.

The solid curves in Fig. 3b show the normalized emission spectra of the red and green blocks, as well as the spectrum of the ultraviolet radiation used for excitation. The dashed curves in Fig. 3b show the absorption spectra of the red and green blocks. To obtain the emission spectra, an ultraviolet laser with 325 nm wavelength (KIMMON KOHA CO., ‘IK3052R-BR') was used to excite the QD blocks. The photoluminescence spectra of the red and green blocks peaked at 629 and 541 nm, respectively. After the inclusion in polymers, the peak emissions of the red and green blocks were shifted towards longer wavelengths by 14 nm and 11 nm, respectively. These red shifts were caused by the subtle energy flow to the polymer. The photoluminescence spectra show similar red shifts; however, the red shifts were barely altered by the emission intensities. On the other hand, each absorption spectrum shows that both the red and green blocks absorbed little visible light.

To fabricate the prototype of the QD-based volumetric display with 8 × 8 × 8 voxels, we followed the steps as follows: (1) We cut each block into 2.5 mm × 2.5 mm × 2.5 mm cubes, which we call ‘QD voxels'. (2) We arrange the QD voxels into a mould in accordance with the layer design shown in Fig. 2b. The eight layers are made separately. (3) We pour a defoamed polymer, which is a mixture of base and curing agent into the mould. (4) We solidify the polymer by heating at 130°C for approximately 3 h; the solidified polymer that includes the QD voxels becomes one of the layers. (5) By stacking the eight layers, we obtain an 8 × 8 × 8-voxel QD-based volumetric display.

**Figure 4:**
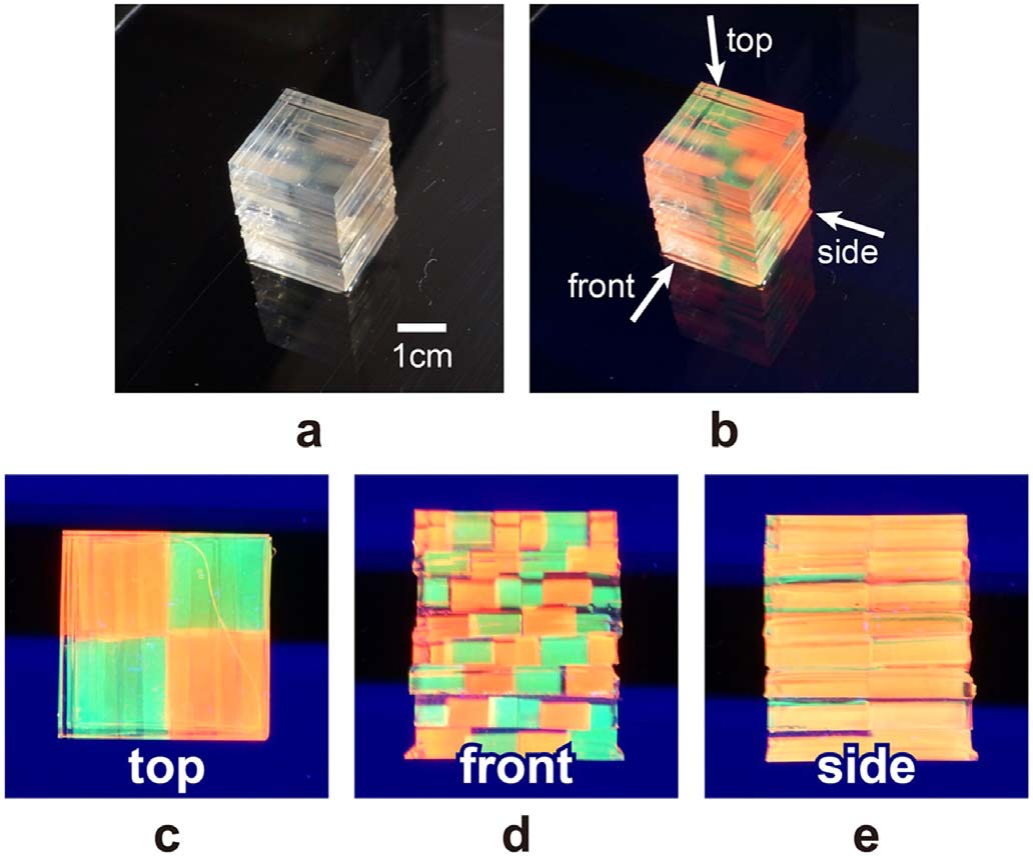
Prototype of QD-based volumetric display. (a) View under natural light. (b) View when excited by ultraviolet light. Patterns when viewed from (c) the top, (d) front and (e) side.

Figure 4 shows the images of the resulting QD-based volumetric display acquired by a CMOS camera. Under white light irradiation, the volumetric display emits essentially no visible light (Fig. 4a). Under ultraviolet irradiation, 3D colour images appear (Fig. 4b). The chequered pattern is visible from the *x*, *y* and *z* directions (Fig. 4c), as are the striped (Fig. 4d) and uniform patterns (Fig. 4e).

**Figure 5:**
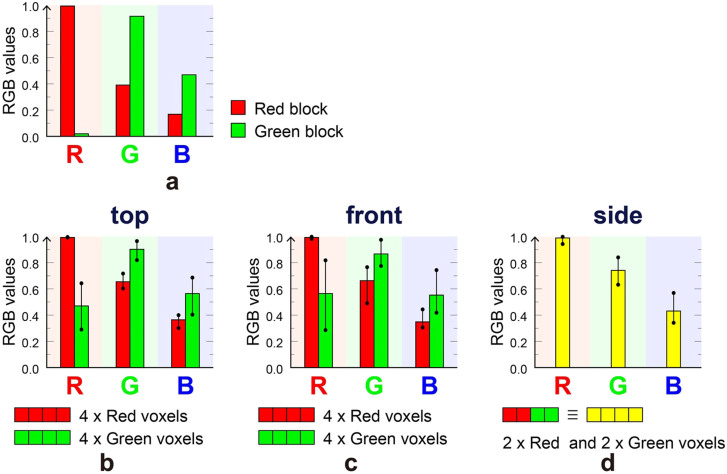
Display quality. RGB values captured by CMOS camera. The labels ‘4 × red voxels' or ‘4 × green voxels' mean that except for four transparent voxels, there are four red voxels or four green voxels out of the eight voxels arranged in depth. (a) RGB values for pure red and green blocks. RGB values are also shown for (b) pattern A (top view), (c) pattern B (front view) and (d) pattern C (side view).

We used the images captured by the CMOS camera to characterize the quality of the images emitted by the volumetric display. Figure 5 shows the average luminance, or the RGB pixel values, of (1) the red and green blocks (Fig. 5a), (2) pattern A (emitted from the top of the volumetric display; Fig. 5b), (3) pattern B (emitted from the front; Fig. 5c) and (4) pattern C (emitted from the side; Fig. 5d). In our design, patterns A and B contain red and green pixels; four red and four green voxels yield a single projected pixel (this is the case for all the red and green pixels). In addition, the yellow pixels in pattern C consist of a combination of two red and two green voxels. Fig. 5a shows that the red and green blocks exhibit high R and G values, respectively. However, with respect to the red pixels of patterns A and B, the G and R values increase and decrease together (see Figs. 5b and 5c). We attribute the increase in the G values to the diffusion of light from the green voxels. The same trend is observed for the green pixels of patterns A and B. In addition, pattern C exhibits higher values for both R and G than the red pixels of patterns A and B, which implies that yellow is obtained by a mixture of light from the red and green voxels. Moreover, we show the maximum and minimum RGB values in Figs. 5b, 5c and 5d.

**Figure 6:**
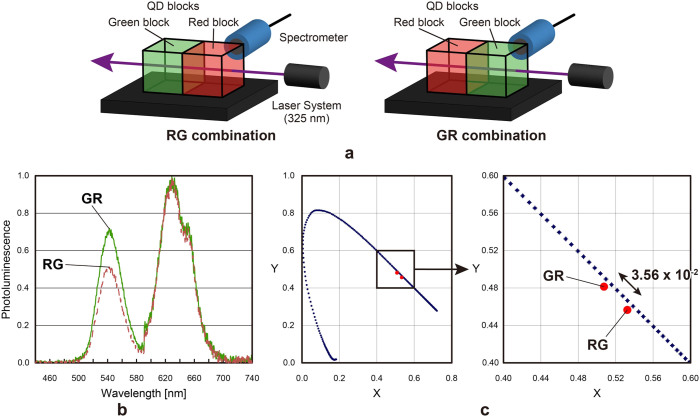
Effect of QD-voxel order on proposed volumetric display. (a) Schematic of the experimental setup. (b) Observed photoluminescence spectra. (c) Effect of QD-voxel order that appears in the chromaticity diagram.

The variance in the pixel RGB values may depend in part on the nonuniformity in the voxel sizes that arises from the fabrication process. However, the mechanism that should also be considered is as follows. As mentioned previously, when the order of the voxels changes with respect to the source of irradiation, the absorption and transmission coefficients of each voxel may cause the colour emitted in a given direction to change. We evaluated such a case by arranging a red block and a green block in series, denoted by ‘RG' (Fig. 6a, left), and for the opposite order, it is denoted by ‘GR' (Fig. 6a, right). The dotted and solid curves in Fig. 6b show the photoluminescence spectra of the RG and GR combinations, respectively. The peak photoluminescence from the green block (541 nm) is shifted by approximately 29% for RG vs. GR, indicating that the emitted colour depends on the order of excitation. Figure 6c shows the spectral difference in a chromaticity diagram. The chromaticity coordinates are calculated on the basis of the colour-matching function defined by the Commission Internationale de l'Eclairage. The distance between the two points on the chromaticity diagram is 3.56 × 10^−2^. Near the chromaticity coordinates of the RG and GR emissions, the human eye cannot differentiate two colours separated by less than approximately 0.5 × 10^−2^ on the chromaticity diagram^13^. Thus, the separation of 3.56 × 10^−2^ between the RG and GR emissions is easily detected by the human eye, and therefore, different colours are observed.

## Discussion

In this study, although we have only used red-light and green-light emitting QDs, QDs that emit other colours are available and can be processed in the same manner as described herein; thus, we can easily increase the types of the QDs used to diversify the available colours. Furthermore, we confirmed that all the QD voxels were in fact excited and emitted light when excited from underneath the volumetric display. This result indicates that occlusions do not need to be considered when arranging the QD voxels. In other words, the proposed QD volumetric display offers high-definition voxels.

We have experimentally demonstrated the principle of presenting multicolour images by combining red and green blocks to obtain a yellow colour. However, the inspection of the image in Fig. 4e shows that the ‘yellow' colour from the side pattern does not obviously differ from the red colour from the top and the front. Notably, the RGB pixel values of the ‘red' colour area of the top (Fig. 5b) and front (Fig. 5c) patterns overlap with those of the side (Fig. 5d) when we consider the error bars. From such a quantitative perspective, the difference between ‘yellow' and ‘red' is not indeed clear. However, to the human eye, the colour of the side pattern is distinctly different from that of the red pixels of the top and front patterns. Note that the average G and B values of the yellow pixels from the side (Fig. 5d) are larger than those of the red pixels from the top (Fig. 5b) and front (Fig. 5c). We attribute the colour difference perceived by the human eye to such increased average G and B values. Therefore, we consider the principle of multicolour to be successfully demonstrated experimentally.

The results also show that the photoluminescence spectra depend on the order in which the QD voxels are excited. The issue of the re-absorption of light emitted from the green QDs by the red QDs is important for the discussion of the order-dependent photoluminescence. For simplicity, we consider the case that a red QD voxel and a green QD voxel are placed close to each other and only the green QDs are excited by ultraviolet irradiation. Here, the sizes of the voxels and the concentrations of QDs are under the same conditions as the experiment. Thereby, 7.1% of green light irradiated from the green QDs is re-absorbed by the red QDs (the ratio of absorption is calculated from the absorbance spectrum.) Under green-light irradiation, the red QDs emit light that is generated from the absorbed light at an efficiency of approximately 32% (the quantum yield is obtained from the certificate of analysis by Sigma Aldrich Co.) Thus, the re-absorption can affect the colour represented by the volumetric display. However, Fig. 6b does not show the difference in the red-light emission between the RG and GR combinations. Although we do not need to consider the effect of the re-absorption for the low-integration scale of the experiment described in this study, an optimal design algorithm for the treatment of the re-absorption will be required for future applications at high definition and high integration.

In principle, the intensity of light emitted from a QD voxel can be adjusted by engineering the QD density for each specific voxel. However, this approach would make fabrication process very complicated. Thus, in future work, we will seek a strategy or a parameter regime that gives an acceptable performance for a given application. Simultaneously, we could also consider exploiting the order dependence of the photoluminescence to obtain different patterns for different viewpoints. Moreover, the excitation wavelength can be tuned to obtain interesting volumetric displays. For example, assume that we select QD_A_ and QD_B_ such that excitation E_1_ excites only QD_A_ and excitation E_2_ excites both QD_A_ and QD_B_. This leads to an additional degree of freedom in the design and enables versatile patterns to be obtained from the QD-based volumetric displays. Furthermore, we will consider the improvement of the implementation process to achieve high definition and high integration. In addition, we will investigate the possibilities of adding further functions by using optical-near-field-mediated energy transfer between the QDs^14^,^15^,^16^,^17^,^18^. For example, a QD mixture of different sizes in a voxel offers increased variety of colours because of the inter-QD energy transfer^19^.

In summary, we demonstrate herein a new QD-based volumetric display. Irradiation by the external light excites the QD voxels and allows multiple patterns to be observed from various viewpoints with no occlusion. By using multiple types of QDs, a multicolour display is obtained.

## Methods

### QD-block preparation

A QD block is fabricated from colloidal QDs and a clear polymer. We used core-shell type CdSe/ZnS QDs (SIGMA-ALDRICH Co., Lumidot™ CdSe/ZnS) dispersed in toluene (5 mg QD per 1 mL toluene). Two types of CdSe/ZnS QDs were used: one with a peak emission at 610 nm and another with a peak emission at 530 nm. For the polymer, we used a SYLGARD 184 Silicone Elastomer Kit by Dow Corning Toray Co., which is made of PDMS and solidified by mixing two liquids (the base and the curing agent) in a ratio of 10:1. The base is a pre-polymer mostly consisting of dimethyl siloxane dimethylvinyl-terminated. The curing agent is primarily made of dimethyl methylhydrogen siloxane trimethylsiloxy-terminated, which yields a cross-linking of the PDMS to cure the pre-polymer by mixing the curing agent with the base. The procedure for making the QD blocks is as follows:

1. Stir 1.1 mL polymer (including 1.0 mL base and 0.1 mL curing agent) and 50 μL (40 μL) of the toluene solution that contains the CdSe/ZnS QDs for the red block (green block).
2. Pour the mixture into a 1 cm cube mould and defoam the mixture overnight under vacuum.
3. Solidify the polymer by heating at 130°C for approximately 3 h.
4. Cut the solidified polymer to form the desired voxel size.

Lumidot™ CdSe/ZnS QDs are surface stabilized with organic ligands (hexadecylamine). Moreover, the QD blocks are expected to be more stable because inclusion in the polymer can prevent the effect of surface oxidation that is the main cause for the QD emission degradation. The emission intensity of a QD voxel may be adjusted by tuning the QD density. The QD density in the QD voxels is determined by the absorbance of the CdSe/ZnS QDs, the extinction coefficient of the CdSe/ZnS QDs and the toluene solution volume. In this study, the red voxels contained approximately 1.6 × 10^12^ of red QDs, and the green voxels contained approximately 9.3 × 10^12^ of green QDs per voxel.

## Author Contributions

R.H., M.N. and T.I. directed the project; R.H., H.N. and M.N. designed the system architecture and the experiments; N.T. and M.O. prepared the QD blocks; R.H., T.K. and N.T. performed optical characterizations; A.S. and T.S. contributed to the discussions.

## Figures and Tables

**Figure 1:**
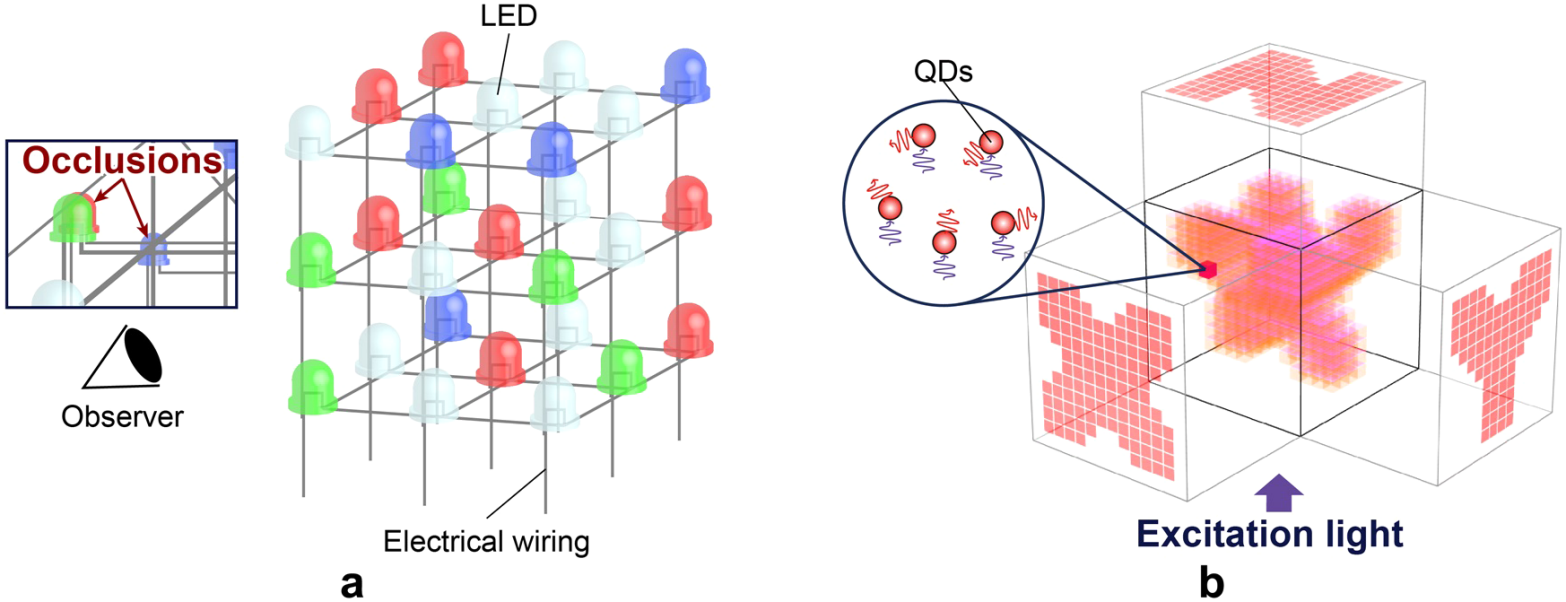
Illustration of the concept of this study. (a) Occlusion occurs when a part of the image viewed from a certain direction is distorted by light scattered from the electrical wiring or other scattering centres. (b) Quantum-dot-based volumetric display exhibits different patterns when viewed from different angles. Each voxel contains quantum dots that are excited by external irradiation.
